# The Understanding of Intentions, Desires and Beliefs in Young Children with Autism Spectrum Disorder

**DOI:** 10.1007/s10803-015-2363-3

**Published:** 2015-01-31

**Authors:** Evelien Broekhof, Lizet Ketelaar, Lex Stockmann, Annette van Zijp, Marieke G. N. Bos, Carolien Rieffe

**Affiliations:** 1 Developmental Psychology, Leiden University, PO box 9555, 2300 RB Leiden, The Netherlands; 2 Center for Autism, Leiden, The Netherlands

**Keywords:** Motivation, Pre-school children, Social behavior, Social cognition

## Abstract

This study provides a comprehensive picture of three core elements (Intentions, Desires, Beliefs) of Theory of Mind (ToM) in young children with autism spectrum disorder (ASD, *n* = 63, *M*age = 55 months) and typically developing children (TD, *n* = 69, *M*age = 54 months). Outcomes showed that ASD and TD children understood intentional actions equally well. Yet, children with ASD lacked the social interest to share intentions. Additionally, children with ASD had more difficulties in understanding others’ desires and beliefs compared to their TD peers. It is discussed whether the ToM delay seen in children with ASD is a motivational or a conceptual problem.

## Introduction

A well-developed Theory of Mind (ToM), the ability to attribute mental states to people and understand their actions based on these mental states, is essential for adaptive social functioning (Dunn 1996). Yet, previous research demonstrates that children with autism spectrum disorder (ASD) show impairments in their ToM development, which might contribute to the explanation of one of the core symptoms: impaired social interaction and communication (Tager-Flusberg 2007).

The ability to ascribe intentions (an action in pursuit of a goal), desires (e.g., hopes, wishes, needs), and beliefs (e.g., thoughts, expectations, convictions) to other people are considered to be key aspects of ToM (Searle 1983). These aspects are intertwined; they all motivate behavior and need to be attributed in order to understand and predict other people’s behavior. The aim of the current study was to simultaneously examine understanding of intentions, desires and beliefs in a group of young children with ASD, compared to a sample of typically developing (TD) peers. Previous studies in children with ASD have often focused on single elements of ToM, and thus our understanding of ToM impairments in children with ASD is still quite fragmented. In other words, there is a lack of research in which all these core elements are examined simultaneously in children with ASD.

Furthermore, significant improvements have been made in the early identification of children with ASD. Earlier, children were rarely diagnosed with ASD before the age of five (Howlin and Moore 1997). Nowadays, this can be done reliably around the age of two (Kleinman et al. 2008). A substantial number of children are diagnosed at age three (i.e., 18 %), and the majority around the age of four (Centers for Disease Control 2012). The improvement in early diagnosis is beneficial for research as it provides the opportunity to investigate children with ASD at a younger age and with relatively larger sample sizes in comparison to earlier studies. This enables examining children with ASD in a more essential period of ToM development, because all its core elements start to develop before the child’s fifth birthday in TD children (Peterson et al. 2005; Colonnesi et al. 2008).

Earlier diagnosis also provides possibilities for studying the early language acquisition in children with ASD and its relation to ToM development. Children with ASD are already found to show lower levels of language competence than TD children around the age of two (Mitchell et al. 2006). The ability to communicate with other people through language is assumed to facilitate ToM development. Children learn about other people’s mental states by for example overhearing their parents talk about what they think or want. Vice versa, ToM skills might also facilitate language acquisition. Being able to understand which object the communication partner is attending to is very helpful in learning the names of objects for example. In TD children as well as in children with ASD, language skills were found to be related to ToM skills (Milligan et al. 2007; Astington and Jenkins 1999; Fisher et al. 2005; Sparrevohn and Howie 1995; Happé 1995), yet most of these studies focused solely on belief tasks as an index of ToM.

This study aims to uniquely contribute to the field of ToM understanding in children with ASD by assessing multiple key elements of ToM simultaneously and examining the relationship between language acquisition and ToM components. As compared to prior research, we will include younger children in a large sample. To ensure diagnostic reliability, we only include children whose diagnoses persisted for 3 years after participation in the study.

### Theory of Mind Development

The order of acquisition of mental concepts follows a certain sequence in typically developing children (Peterson et al. 2005; Wellman and Liu 2004). The understanding of intentions starts to develop first and is therefore usually examined when interested in the earliest roots of ToM development (Meltzoff 1995; Camaioni et al. 2004). Subsequently, the capacity to understand desires precedes the capacity to understand beliefs (Wellman and Liu 2004).

This progressive order has been found to be identical in children with ASD. Only, the latter group seems to be delayed in age of attainment in some stages (Peterson et al. 2005). The following sections will therefore discuss the development of understanding intentions, desires and beliefs separately for children with ASD compared to TD children.

### Intention Understanding

Intention understanding involves the acknowledgement that physical action depends on the goals and intentions of an actor. Children first start to understand the basics of this intentional action, before they are able to respond to others’ intentions to require or share something. This latter ability also requires a motivation to share intentions socially (Tomasello et al. 2005).

Research in the understanding of intentional action indicates that nine-month-old infants already comprehend that actions are based on intentions. These young infants can distinguish between purposeful and accidental actions. In one study the experimenter played a game in which toys were handed to the child across a table (Behne et al. 2005). The nine-month-old infants showed more impatience when the experimenter was unwilling to give them the toy than when s/he was unable to do so. Intentional action understanding also involves making goal references beyond observed events. Meltzoff (1995) showed that eighteen-month-olds were able to complete an unseen goal after seeing an adult demonstrate an act but failing to achieve this end goal.

Several studies examined the understanding of intentional action in children with ASD and reported inconsistent results depending on the tasks used. One study showed that it was more difficult for adolescents with ASD to acknowledge that an action was accidental compared to TD four-year-olds (Phillips et al. 1998). However, this finding was not replicated in a study by Russell and Hill (2001). Two other studies used versions of Meltzoff’s (1995) experiment and also did not find impairments in intention understanding in children with ASD between the ages of 2 and 5 years (Carpenter et al. 2001; Aldridge et al. 2000).

After developing the understanding that actions are intentional, TD children also start to respond to others’ intentions by directing their attention and communication around the age of one (Camaioni et al. 2004). At this age, TD children can locate a specific target following an adult’s pointing gesture. This ability for joint attention refers to the process in which two individuals share visual attention for the same external object or event (Tomasello et al. 2005). Literature distinguishes two types of pointing gestures which differ in their underlying motive: imperative and declarative pointing. Imperative comprehension refers to understanding that the other is requesting an object by pointing to it, whilst declarative comprehension refers to understanding that the other is directing attention with the sole motivation to share attention for the same object or event (Bates et al. 1975; Carpenter et al. 2001).

The acquisition of declarative comprehension contributes to language development. Declarative comprehension establishes shared attention for the same stimulus in, for example, a child and a caregiver. Language used by the caregiver is usually related to the particular event, and thereby fosters word learning (Mundy et al. 2007). Indeed, declarative comprehension early in life has been related to a higher level of language competence in the later development of TD children (Kristen et al. 2011).

Studies have found that children with ASD are less inclined than TD children to use pointing gestures themselves [see review by (Bruinsma et al. 2004)], and also less frequently respond to pointing gestures or the eye gaze of others (e.g., Leekam and Ramsden 2006; Dawson et al. 2004). Major deficits in responding to bids for joint attention are considered one of the earliest signs of ASD (Murray et al. 2008). This pervasive unresponsiveness is so frequently observed that it is actually included as a diagnostic criterion (DSM 5; American Psychiatric Association 2013). Interestingly, it has been found that children with ASD are impaired with regard to the comprehension of declarative pointing but not in imperative pointing (Baron-Cohen 1989).

### Desire Understanding

TD children as young as 2 years of age can predict someone’s behavior based on the desires of that person. For example, in a study by Wellman (1990), two-year-old children were told that a story character enjoys swimming. When children were asked whether this character would go swimming or go to the park, children were able to correctly predict the subsequent act. This indicates that children understand that desires motivate behavior. Yet, this does not necessarily imply that children understand the subjectivity of desires. What if children in the Wellman study hated swimming themselves? Would they still have predicted the story character would go swimming? Subsequent research suggests they would not have succeeded in that case, because children of two years of age let their own desires guide their predictions of the behavior of others. Around the age of four TD children acknowledge the subjective character of desires (Rieffe et al. 2001).

Previous studies indicate that the understanding of desires in children with ASD is in line with their mental age (Baron-Cohen 1991). Children with ASD often show an adequate understanding of desires as inner drives which cause behavior (Phillips et al. 1995; Peterson et al. 2005). However, these studies have not controlled for the child’s own preferences and it is therefore unclear whether children with ASD would also attribute desires to others which differed from their own. Therefore, to date, it is still inconclusive whether children with ASD truly appreciate the subjectivity of desires.

### Belief Understanding

The development of belief understanding begins slightly later than desire understanding, with the notion that beliefs govern actions (Peterson et al. 2005). Subsequently, children also start to acknowledge the subjectivity of beliefs, which is often measured with the traditional false belief task. In this task children are presented with a story in which one character has a belief about a location of an object that does not correspond to the real location. Then, children are asked where this character will look for the object. TD children around the age of four successfully predict that the character will look for the object at the location where s/he thinks the object is, instead of the real location (Wimmer and Perner 1983; Wellman et al. 2001).

Difficulties in understanding false beliefs in children with ASD have received a great amount of attention. Baron-Cohen et al. (1985) found that 80 % of the children with ASD failed the false belief task, even though they had a verbal mental age above 5 years old. A large number of studies have replicated this finding and have indicated that the majority of children with ASD pass false belief tasks when they have a verbal-mental age of at least 11 years (for a review see Happé 1995).

### Current Study

In this study, we aimed to investigate three core elements of ToM in two- to six-year-old children with ASD compared to TD children. For intention understanding, we hypothesized that children with ASD understand intentional actions to the same extent as their TD peers (Aldridge et al. 2000; Carpenter et al. 2001). Additionally, we expected no difference in responses between the two groups with regards a pointing gesture carried out by the experimenter, requesting an object (i.e., imperative comprehension).Yet, we did expect fewer responses from the children with ASD to a pointing gesture, which is solely produced in order to share attention (i.e., declarative comprehension), compared to their TD peers (Baron-Cohen 1989).

For desire understanding, we expected children with ASD to predict behavior successfully based on desires when these desires corresponded with their own desires (i.e., Similar desires) (Phillips et al. 1995). However, we expected that the children with ASD would find it more difficult to predict the behavior of others, when that desire was in conflict with their own desire (i.e., Dissimilar desire). As repeatedly suggested in the literature, we expected children with ASD to be less able to understand false beliefs when compared with TD children (Baron-Cohen et al. 1985; Happé 1995).

We also aimed to explore the relationship between declarative comprehension and language competence. We expected to find a positive relationship in both children with ASD and TD, because both concepts have been related before in TD children. Confirmation of this hypothesis might explain language difficulties often seen in children with ASD (Kristen et al. 2011).

## Methods

### Participants and Procedure

In total, 150 children between the ages of 2 and 6 years participated in this study. The sample included 78 children with ASD recruited via an institution specialized in diagnosing ASD in children and adolescents: the Center For Autism in Leiden, the Netherlands. Children were recruited in two ways: (1) Parents of children who had already received a diagnosis within the autistic spectrum were approached; (2) Parents of children who were still in the diagnostic process were contacted. Only those children who received a formal diagnosis were included in the sample. A diagnosis within the autistic spectrum (i.e., autistic disorder, Asperger’s disorder, PDD-NOS) was issued using the DSM-IV-TR criteria by a qualified child psychologist or psychiatrist using parental reports and clinical observation. Three years later, families were contacted to investigate whether children had retained their diagnostic status over time. In the ASD group 62 children had maintained their diagnosis (79.5 %), 14 children moved from the autistic spectrum (17.9 %), and the parents of 2 children could not be contacted (2.6 %).

The sample also included 72 TD children, recruited from day-care centers and mainstream primary schools. Parents and/or teachers indicated that TD children were free of any clinical problem. The TD children were matched with the children with ASD based on age and gender. Like the ASD group, families were contacted to investigate whether children were still free of clinical problems. In the TD group, one child had received an ASD diagnosis in the meantime, and two children were excluded because they had developed a non-autistic developmental disorder. This leaves a sample of 63 children with ASD (Mean age = 54 months, SD = 12.7) and 69 TD children (Mean age = 55 months, SD = 14.4).

TD children had been tested by the SON-R (a standard Dutch non-verbal intelligence test), and IQ scores from children with ASD were retrieved from school files or tested at the Centre for Autism. Children with ASD were therefore tested using various IQ tests (i.e., SON-R, WISC III, WPPSI and WNV-NL). Only children with an IQ above 70 were included in the study. IQ scores were missing for 21 TD children and 7 children with ASD. TD children had a higher IQ score compared to children with ASD, *t*(102) = 3.25, *p* = .002, *r* = .31. Table 1 shows descriptive characteristics for both samples.

**Table 1:** Characteristics of participants

	ASD (*n* = 63*)*	TD (*n* = 69)
IQ score, mean (SD)*	99.9^b^	110.0^a^
Age, mean (SD), mo	54.6 (12.7)	54.5 (14.4)
Age, range, mo	21–72	21–72
Gender, no. (%)		
Male	55 (87)	60 (87)
Female	8 (13)	9 (13)
ASD subtype, no. (%)		
Autistic disorder	39 (62)	
PDD-NOS	24 (38)	
Age at diagnosis, no. (%)		
1 year	1 (2)	
2 years	5 (8)	
3 years	11 (18)	
4 years	15 (23)	
5 years	14 (22)	
Unknown	17 (27)	

IQ scores were missing for 7 children with ASD and 21 TD children

Different letter-superscripts indicate differences on rows at *p* < .05

The Ethics committee of Leiden University and the Center for Autism granted permission for the study and all parents gave written consent before testing. All children were tested individually in a quiet room at home, school, or at the Center for Autism. Sessions took ~30 min.

## Materials

### Indices for Language

The *Child Development Inventory* (CDI; Ireton and Glascoe 1995) assesses the current level of development of 1–6 year-olds. In this study we used 2 scales of this questionnaire: Expressive language (50 items) and Language comprehension (50 items). For each item the parent is presented with a statement and asked to indicate whether this does or does not apply to their child (0 = no, 1 = yes). Both scales showed excellent reliability, with a Cronbach’s alpha of .98 for Expressive language and .97 for Language comprehension.

Although the desire and belief tasks were designed to place minimal verbal demand on children, they did involve a short story. To ensure task comprehension, the tasks were only administered to children with sufficient language skills (Ketelaar et al. 2012). To establish whether children would be able to understand the short stories used in the tasks, we assessed whether children could comprehend short sentences and whether they were familiar with the objects used in the stories. First, parents were asked if their children understood a series of simple sentences. These sentences matched the structure of the ones used to formulate stories in the desire and belief tasks. Second, children were shown a page with the 13 objects present in the desire and belief task stories. The experimenter named the objects individually and children were instructed to point to the corresponding object. None of the children who—according to their parents—understood simple sentences made more than two mistakes when pointing to the named objects. These children were deemed to have sufficient language skills (see Table 2 for an overview of children with sufficient and insufficient language skills).

**Table 2:** Mean scores on age, language comprehension and language expression as a function of group by language-comprehension skills

	Sufficient language comprehension		Insufficient language comprehension	
	ASD (*n* = 45)	TD (*n* = 62)	ASD (*n* = 18)	TD (*n* = 7)
	Mean (SD)	Mean (SD)	Mean (SD)	Mean (SD)
Age, mo	59.1^a^ (8.22)	57.8^a^ (10.80)	43.2^b^ (14.88)	24.4^c^ (2.99)
CDI, Language comprehension (0–1)	0.82^b^ (0.15)	0.93^a^ (0.10)	0.43^c^ (0.31)	0.39^c^ (0.24)
CDI, Expressive language (0–1)	0.86^b^ (0.13)	0.95^a^ (0.09)	0.46^c^ (0.31)	0.43^*c*^ (0.12)

Different letter-superscripts indicate differences on rows at *p* < .05

### Indices for Intention Understanding

The “Intention Understanding task” (Ketelaar et al. 2012; Meltzoff 1995) examines children’s understanding of the intentions of others in performing a specific action. The experimenter acted out 3 separate intentions but failed to achieve the final goal state: dropping a string of beads in a cup, sliding a tube in a slightly wider tube and stacking 2 cups. For each intention, the experimenter made 3 attempts and then handed the material to the child. The children passed this task if they completed the intention and they received 1 point for each produced target act (range 0–3).

In the “Imperative Comprehension task” (Colonnesi et al. 2008; Ketelaar et al. 2012) the experimenter pointed to an object which was beyond the experimenter’s but within the children’s reach. Then, the experimenter requested the object by holding out her hand and alternating between looking at the child and the object. Children passed this task if they gave the object to the experimenter, put the object on the table near the experimenter, or refused to do so (e.g., saying ‘no’). The pointing gesture was alternated with other tasks and repeated until children passed, up to a maximum of 3 attempts. Children could earn 3 points if they produced the target behavior the first time, 2 points if they produced it the second time, and 1 point if they produced it the third time.

In the “Declarative Comprehension task” (Colonnesi et al. 2008; Ketelaar et al. 2012) the experimenter pointed in surprise toward a stimulus which stood just behind the child, but at his/her eye level. Then, the experimenter alternated between looking at the child and the stimulus and waited passively for a subsequent 10 s. Children could earn 1 point for each of the following behaviors: looking at the stimulus, looking at the experimenter, and making an attempt to communicate (e.g., pointing or vocalizing) about the object (range 0–3).

Eight children had missing data on one of the intention tasks and were therefore not included in the analyses.

### Indices for Desire Understanding

In the “Desire task,” (Ketelaar et al. 2012) the child was presented with 4 vignettes which were each supported by pictures. First, a picture was shown in which 2 food items were depicted (e.g., candy and sandwich). Children were asked which food item they liked best. Second, a boy was introduced into the picture story. In 2 vignettes, the boy had a preference that corresponded to the child’s preference; the Similar condition. In the other 2 vignettes, the preference of the boy conflicted with the child’s preference; the Dissimilar condition. After the vignettes were presented, children were asked: “Which food will the boy choose?” To make sure that children understood the vignette and had memorized the information correctly 2 control questions were asked regarding the boy’s preferences (e.g., “Does the boy like candy/sandwich?”). To earn 1 point, children were required to answer the test question and control questions correctly. Children were given 0 points if they failed to answer the test question or one or more control questions. Mean scores were calculated for the Similar and Dissimilar task separately.

### Indices for Belief Understanding

The “False Belief task” (Ketelaar et al. 2012) follows the same procedure as the Sally–Ann task described in Baron-Cohen et al. (1985). Children were presented with a picture story in which a boy puts a toy in one location and leaves the scene. While he is gone, a girl moves the toy to another location. Then, the boy returns and wants to play with his toy. Children were asked: “Where will the boy look for his toy?” In addition, 2 control questions were asked: “Where is the toy now?” and “Where did the boy put the toy before he went away?” Children could earn 1 point if they answered all questions correctly. Children who failed to answer one of the questions received 0 points. When they did not respond or failed to answer verbally to one of the questions children were treated as missing (9 ASD, 3 TD).

## Results

### Intention Understanding

The mean scores of all of the ToM tasks (intentions, desires, and beliefs) are shown in Table 3. Children’s intention understanding was examined, using a 2 (Group: ASD, TD) × 3 (Task: Intention Understanding, Imperative Comprehension, Declarative Comprehension) mixed analysis of variance, which produced a main effect for Group, *F*(1, 122) = 10.11, *p* = .002, $\upeta_{\text{p}}^{2} = .08$, which was qualified by a Group × Task interaction, *F*(2, 244) = 3.29, *p* = .039, $\upeta_{\text{p}}^{2} = .03$. Mean scores revealed that children with ASD scored lower than the TD children on imperative [*t*(122) = 2.86, *p* = .005, *r* = .25] and declarative comprehension [*t*(122) = 3.31, *p* = .001, *r* = .29], but not in understanding intentional acts [*t*(122) = .08, *p* = .934, *r* = .01].

**Table 3:** Mean scores on intention, desire and belief tasks as a function of group by task

Instrument (min–max)	ASD	TD	Between-group difference (95 % CI)
	Mean (SD)	Mean (SD)	
	*n* = 56	*n* = 68	
Intention-understanding (0–3)	2.30^a^ (0.99)	2.31^a^ (0.91)	0.01 (−.33, .35)
Imperative comprehension (0–3)	2.09^a^ (1.16)	2.60^a^ (0.83)	0.51* (.16, .87)
Declarative comprehension (0–3)	1.88^b^ (1.10)	2.38^a^ (0.57)	0.51* (.20, .81)
	*n* = 45	*n* = 62	
Similar desire (0–1)	0.72^a^ (0.39)	0.86^a^ (0.31)	0.13 (−.01, .27)
Dissimilar desire (0–1)	0.51^b^ (0.46)	0.83^a^ (0.35)	0.32* (.16, .48)
	*n* = 36	*n* = 59	
False belief (0–1)	0.42 (0.50)	0.66 (0.48)	0.24* (.04, .45)

Different letter-superscripts indicate differences on columns at *p* < .05

Additionally, we also analyzed intention understanding with IQ score as a covariate. Both the main effect for Group, *F* (1, 94) = 10.99, *p* = .001, $\upeta_{\text{p}}^{2} = .11$, and the Group × Task interaction remained significant, *F*(2, 188) = 3.23, *p* = .042, $\upeta_{\text{p}}^{2} = .03$.

Exploratory analysis was conducted in order to investigate whether children with ASD were less responsive to imperative bids for joint attention altogether, or just needed more bids before they responded. In this additional analysis, children in the imperative comprehension task received 1 point if they responded to at least one bid for joint attention, irrespective of the number of trials needed, and received 0 points if they failed to respond to all three trials. According to this scoring procedure no differences were found in the performance of ASD and TD children, *t*(127) = 1.85, *p* = .067, *r* = .16.

### Language Skills

Within our sample, 18 children with ASD and 7 TD children had insufficient language-abilities, according to the criteria described in the materials section. One-way ANOVA’s with Bonferonni correction showed that children with ASD and TD children with sufficient language abilities were older than their peers without this required ability, *F*(3, 128) = 31.59, *p* < .001, η^2^ = .43 (see Table 2). Children with ASD with sufficient language abilities did not differ in age from TD children with sufficient language ability.

A somewhat different pattern was observed when language-comprehension was analyzed, as scored by parents, on the CDI questionnaire. A one-way ANOVA revealed that TD children with sufficient language skills were scored higher on language-comprehension than children with ASD with sufficient language skills, and children of both groups without sufficient language skills had the lowest scores, *F*(3, 105) = 43.66, *p* < .001, η^2^ = .56 (see Table 2). The same pattern was observed for language expression scores given by parents on the CDI questionnaire, *F*(3, 105) = 47.03, *p* < .001, η^2^ = .57 (see Table 2).

### Desire Understanding

Only children with sufficient language skills were included in a 2 (Group: ASD, TD) × 2 (Task: Similar Desire, Dissimilar Desire) mixed analysis of variance. This analysis showed main effects for Group, *F*(1, 105) = 14.38, *p* < .001, $\upeta_{\text{p}}^{2} = .12$, and Task, *F*(1, 105) = 7.79, *p* = .006, $\upeta_{\text{p}}^{2} = .07$, which was qualified by a Group × Task interaction, *F*(1, 105) = 4.92, *p* = .029, $\upeta_{\text{p}}^{2} = .05$. Post hoc *t* tests showed that the TD children outperformed children with ASD on the Dissimilar Desire task [*t*(105) = 4.09, *p* < .001, *r* = .37] but not on the Similar Desire task [*t*(105) = 1.97, *p* = .052, *r* = .19]. In addition, children with ASD had lower scores on the Dissimilar task compared to the Similar task, *t*(44) = 2.74, *p* = .009, *r* = .38. This difference was not seen in the TD group, *t*(61) = .54, *p* = .594, *r* = .07 (see Table 3).

In a mixed analysis of covariance which corrected for IQ, the main effect for group, *F*(1, 90) = 21.87, *p* < .001, $\upeta_{\text{p}}^{2} = .20$ and Task *F*(1,90) = 5.16, *p* = .025, $\upeta_{\text{p}}^{2} = .05$ remained, but the Group × Task interaction effect was no longer significant, *F*(1, 90) = 3,47, *p* = .066, $\upeta_{\text{p}}^{2} = .04$. These two main effects illustrated that TD children outperformed children with ASD; and both groups scored higher on the Similar than the Dissimilar Desire task.

### Belief Understanding

Children with ASD performed less well on the false belief task than TD children, *t*(93) = 2.38, *p* = .019, *r* = .24 (see Table 3). In an analysis of covariance with IQ as covariate, the main effect for Group remained significant, *F*(1, 80) = 9.60, *p* = .003, $\upeta_{\text{p}}^{2} = .11$.

### ToM Abilities and Language

Table 4 shows correlations of IQ with declarative comprehension, desire and belief understanding for both groups separately. Performance on the Similar and Dissimilar Desire task were both related to IQ in TD children, whereas in the ASD group IQ was only related to the performance on the Dissimilar desire task. No other relationships with IQ were found.

**Table 4:** Correlation coefficients (partial correlations corrected for age) of declarative comprehension and ToM tasks with IQ, age and language

	ASD				TD			
	IQ	Age	CDI, LC	CDI, EL	IQ	Age	CDI, LC	CDI, EL
	*n* = 52	*n* = 58	*n* = 53	*n* = 53	*n* = 48	*n* = 69	*n* = 51	*n* = 51
Declarative comprehension	.00	.34^*^	.23 (−.04)	.23 (−.03)	−.27	−.01	.23 (.45^**^)	.22 (.38^**^)
	*n* = 45	*n* = 45	*n* = 43	*n* = 43	*n* = 48	*n* = 62	*n* = 47	*n* = 47
Similar desire	.05	.24	.20 (.03)	.27 (.15)	.37^**^	.55^***^	.62^***^ (.35^*^)	.31^*^ (−.28)
	*n* = 45	*n* = 45	*n* = 43	*n* = 43	*n* = 48	*n* = 62	*n* = 47	*n* = 47
Dissimilar desire	.34^*^	−.07	−.02 (.05)	.11 (.24)	.34^*^	.37^**^	.45^**^ (.27)	.44^**^ (.25)
	*n* = 36	*n* = 36	*n* = 35	*n* = 35	*n* = 47	*n* = 59	*n* = 44	*n* = 44
False belief	.25	.29	.16 (−.10)	.32 (.17)	−.26	.37^**^	.46^**^ (.30)	.37 ^**^ (.14)

*CDI* child development inventory, *LC* language comprehension, *EL* expressive language

*p < .05; **p < .01; ***p < .001 (2-tailed)

In addition, correlations of declarative comprehension with age, language comprehension and expressive language were computed for both groups separately. Within the ASD group, declarative comprehension was related with age but this was not the case in the TD group. After correcting for age, declarative comprehension was significantly related both to language comprehension and to expressive language in TD children, but not in children with ASD (Table 4).

To assess the relationships between desire and belief understanding with age, expressive language and language comprehension, we computed correlations for both groups separately. Also partial correlations, corrected for age were calculated. Age correlated with all desire and belief tasks for the TD group, but not for the ASD group. Both language skills correlated with all ToM abilities in children with TD, but again not for the ASD group. When corrected for age, only the correlation between language comprehension and the Similar Desire task remained significant in the TD group (see Table 4).

## Discussion

The aim of the present study was to gain a better understanding of three core elements of ToM skills in young children with ASD. Our study confirms previous studies which demonstrated that young children with ASD (Mean age 55 months) can understand other people’s intentional acts to the same extent as their TD peers, because children in both groups could equally often finish the experimenter’s failed acts (Aldridge et al. 2000; Carpenter et al. 2001). Despite this promising outcome, we did observe lower performances in children with ASD when compared to their TD peers when intention understanding involved social sharing, as is the case in both the imperative and declarative pointing comprehension. Additionally, children with ASD and TD children performed equally well when predicting the choices of others based on the protagonist’s desires, but when the desires conflicted, children with ASD more often attributed their own desire to the protagonist than did their TD peers. This pattern was also evident when we tested their false belief understanding; children with ASD more often predicted the story character’s behavior based on their own belief.

These findings remained mostly unchanged when IQ was taken into account except for children’s scores on the desire tasks. When IQ was controlled for, children with ASD scored lower than their TD peers on both desire tasks. Possibly, the desire task also did a stronger appeal on other cognitive functions, such as short term memory or verbal abilities. Nevertheless, both groups still performed better on the Similar than the Dissimilar desire task as was expected, showing that children of this age acknowledge that desires guide behavior, but not necessarily that different people can have different desires which guide their actions (Rieffe and Terwogt 2000).

### Language

In line with the literature, we found a positive relationship between declarative comprehension and both language comprehension and expression in the TD group (Kristen et al. 2011; Astington and Jenkins 1999). Unexpectedly and contrary to previous studies (Fisher et al. 2005; Happé 1995), these concepts were not related in the ASD group. A possible explanation for this contrary finding is that children in our sample were younger than in prior research examining this relationship. Factors other than declarative comprehension might play a more pronounced role in the acquisition of language in children with ASD. A cautious interpretation is recommended, because while it has been indicated that language comprehension and expression can both be measured reliably by parent report, our findings rely on the CDI, which is not a formal test of language abilities (Ireton and Glascoe 1995). Future studies should unravel which factors are important in the early language learning of children with ASD.

### Measuring Intentional States

In the present study, children with ASD and TD children were equally capable of finishing the experimenter’s failed acts, which led us to conclude that the ability to derive intentions from behavioral acts was intact in the ASD group. It bears mentioning that other studies have questioned whether performance on this task, as developed by Meltzoff (1995), truly reflects acknowledgement of intentions rather than desires (Williams and Happe 2010). Indeed, intentions and desires are difficult to disentangle since they both reflect intentional states which are aimed at ‘the world to fit the mind’, preventing us from ruling out that performance on Meltzoff’s task also partly reflects children’s desire understanding. However, desires are met when they are fulfilled, whereas intentions are met when carried out (Searle 1983). Therefore, we wish to argue that the current task, in which the child is expected to finish a previously unknown, yet unfinished action by the experimenter, undoubtedly reflects intention understanding, but not necessarily children’s desire understanding.

In addition, is has been argued that intention understanding cannot be measured reliably as fully-fledged understanding of intentions only emerges at a lager age (Williams and Happe 2010). Nevertheless, we think that it is necessary and important to examine the early signs of this development, especially in clinical groups which are known for their impaired development. The earlier we can detect different pathways in development compared with TD children, the better professionals can tailor their interventions.

### Social Sharing

Previous research suggests that declarative comprehension is impaired in children with ASD compared to TD children, while imperative comprehension is assumed to be intact (Camaioni 1997, 2004; Baron-Cohen 1989). To our surprise, children with ASD in our study not only had difficulty in declarative comprehension, but also in imperative comprehension compared to TD children. Imperative comprehension and declarative comprehension are not more complex than the comprehension of intentional action. Yet, these tasks do differ on one important aspect: both imperative and declarative comprehension requires the motivation and skills for sharing psychological states with others (Tomasello et al. 2005). This requirement is often not met by children with ASD, who display a lack of interest in social communication (American Psychiatric Association 2013). Based on this knowledge, a lower response to both imperative and declarative pointing gestures might not come as a surprise in children with ASD compared to TD children.

The design of the imperative comprehension task in our study enabled us to examine whether the lower performance of the ASD group on this task represented an inability or a lack of social interest. A lower score on imperative comprehension indicated that children with ASD needed more trials to understand that the experimenter was requesting a certain object, but this does not necessarily imply that these children are not able to understand the request. Indeed, when we only scored whether children passed or failed, irrespective of the amount of trials, children with ASD do no longer perform lower compared to TD children. These findings might indicate that the lower performance on imperative comprehension of children with ASD could have been a reflection of lower motivation to share intentions than TD children, rather than an inability to comprehend the experimenters’ intention.

Our suggestion that lower ToM performance may be a reflection of lower social motivation in children with ASD could also be extended to the desire and false belief tasks. This would be congruent with other studies in which task motivation was manipulated (Begeer et al. 2003, 2006). In a study by Begeer et al. (2003), two false belief tasks were administered, and children were told they would be rewarded for only one of these tasks with candy. Children with ASD tended only to correct false beliefs when rewarded with the candy, which indicates that they are able to understand false beliefs when they are externally motivated. Therefore, it could be questioned whether the ToM performance of the children with ASD in our sample could also be increased when they are externally motivated. This question is particularly important for early interventions, because it indicates that ToM abilities are present but not automatically activated in children with ASD. The conditions under which task motivation is enhanced and results in increased ToM performance should be explored.

### Diagnostic Stability

Despite the benefits of early confirmation of ASD in children, early diagnosis also has a major disadvantage for clinical practice, as well as for research: an initial diagnosis before the age of five is not always retained. For example, one prospective study indicated that according to clinical judgment, nineteen percent of the children diagnosed with ASD between 16 and 35 months moved off the autistic spectrum by the second evaluation in later childhood (Kleinman et al. 2008). These findings could be caused by the difficulty to distinguish children with ASD at this age from children with severe global developmental delay (Lord 1995). However, the inclusion of these children in research samples might have influenced earlier findings regarding ToM abilities in young children with ASD. In the present study, we partly overcame this problem by only including children with ASD who retained their diagnosis for 3 years. Yet, not all children with ASD in our sample were formally reassessed consistently after 3 years by qualified professionals, For future studies, we would suggest adopting this approach in order to better distinguish children with ASD from children with a several global developmental delay.

## Conclusion

This study may indicate that children with ASD do understand intentional action but lack the social interest to share intentions with others. These findings strongly suggest that children with ASD do not seem to appreciate the subjective character of both desires and beliefs.

Since the motivation to share intentions was not directly measured in our study, we cannot state with certainty that the difference in sharing intentions between the TD and ASD group can be derived to the motivation to share intentions. Future studies are needed in order to examine the role of social motivation in ToM functioning. When lower ToM performance in research does indeed reflect a lack of social interest, as we hypothesized, interventions should be aimed at making perspective taking abilities more rewarding during the essential developmental period. A better understanding is needed regarding the influence of the separate core elements on later social functioning
